# Cenobamate: real-world data from a retrospective multicenter study

**DOI:** 10.1007/s00415-024-12510-1

**Published:** 2024-07-01

**Authors:** Stephan Lauxmann, David Heuer, Jan Heckelmann, Florian P. Fischer, Melanie Schreiber, Elisabeth Schriewer, Guido Widman, Yvonne Weber, Holger Lerche, Michael Alber, Sigrid Schuh-Hofer, Stefan Wolking

**Affiliations:** 1 Department of Neurology and Epileptology, Hertie Institute for Clinical Brain Research, University of Tuebingen, Hoppe-Seyler-Str. 3, 72076 Tuebingen, Germany; 2 Department of Epileptology and Neurology, RWTH Aachen University Hospital, Aachen, Germany; 3 Department of Pediatric Neurology and Developmental Medicine, University Children’s Hospital, Tuebingen, Germany

**Keywords:** Cenobamate, Drug-resistant epilepsy, Anti-seizure medication, Adverse drug reactions

## Abstract

**Background:**

Clinical trials have shown that cenobamate (CNB) is an efficacious and safe anti-seizure medication (ASM) for drug-resistant focal epilepsy. Here, we analyzed one of the largest real-world cohorts, covering the entire spectrum of epilepsy syndromes, the efficacy and safety of CNB, and resulting changes in concomitant ASMs.

**Methods:**

We conducted a retrospective observational study investigating CNB usage in two German tertiary referral centers between October 2020 and June 2023 with follow-up data up to 27 months of treatment. Our primary outcome was treatment response. Secondary outcomes comprised drug response after 12 and 18 months, seizure freedom rates, CNB dosage and retention, adverse drug reactions (ADRs), and changes in concomitant ASMs.

**Results:**

116 patients received CNB for at least two weeks. At 6 months, 98 patients were eligible for evaluation. Thereof 50% (49/98) were responders with no relevant change at 12 and 18 months. Seizure freedom was achieved in 18.4% (18/98) at 6 months, 16.7% (11/66), and 3.0% (1/33) at 12 and 18 months. The number of previous ASMs did not affect the seizure response rate. Overall, CNB was well-tolerated, however, in 7.7% (9/116), ADRs led to treatment discontinuation. The most frequent changes of concomitant ASMs included the discontinuation or reduction of sodium channel inhibitors, clobazam reduction, and perampanel discontinuation, while brivaracetam doses were usually left unchanged.

**Conclusions:**

CNB proved to be a highly effective and generally well-tolerated ASM in patients with severe drug-resistant epilepsy, comprising a broad array of epilepsy syndromes beyond focal epilepsy.

## Introduction

Epilepsy affects approximately 1% of people and is one of the most burdensome chronic neurological diseases [1]. Despite the introduction of more than 20 anti-seizure medications (ASMs) in the last 30 years, approximately one-third of patients remain drug-resistant [2]. The most significant burden of drug resistance occurs in focal epilepsies, with a prevalence of drug resistance beyond 50% [3]. As of today, epilepsy surgery represents the only curative therapy strategy, but only a tiny fraction of patients undergo presurgical evaluation and turn out to be a suitable surgery candidate [4]. Thus, the majority of drug-resistant cases rely on the optimization of the drug regime, underlining the need to develop more effective ASMs.

Cenobamate (CNB) is one of the most recent ASMs to receive approval by licensing authorities in the US and Europe as add-on treatment in adults with focal seizures and with focal to bilateral tonic–clonic seizures that have not been sufficiently controlled with at least two previous ASMs. CNB retains a dual mechanism of action by inhibiting persistent and transient sodium currents of voltage-dependent sodium channels and acts as a positive postsynaptic allosteric modulator of gamma-aminobutyric acid (GABA_A_) receptors [5–7].

The effectiveness of CNB has been shown in a multicentric, randomized, placebo-controlled trial in patients with focal seizures who were not sufficiently controlled with one to three concomitant ASMs [8]. Daily doses of CNB ranged from 100 to 400 mg/day. Seizure freedom rates, i.e., 100% seizure reduction in a 12-week observation interval, were dose-dependent and occurred between 3.9% (100 mg daily) and 21.1% (400 mg daily) of study participants. Likewise, response rates, i.e., > 50% seizure reduction from baseline, ranged from 40.2% (100 mg daily) to 64.2% (400 mg daily). Open-label extension studies found seizure freedom rates of between 13.1 and 16.4% and > 50% seizure reduction of between 71.1 and 76.1% compared to baseline [9, 10]. During early clinical development with high initial treatment doses of 50 or 100 mg daily, three cases of drug reaction with eosinophilia and systemic symptoms (DRESS) occurred, including one fatality [8]. Later studies featuring a lower starting dose of 12.5 mg and biweekly increments with a safety population of 1,339 patients did report no cases of DRESS [9].

Lately, several real-world studies have confirmed the overall high efficacy and favorable safety and tolerability profile of CNB [11–15]. Here, we present one of the largest real-world studies to assess safety, tolerability, efficacy, and ASM combinations in a two-center cohort of 116 patients with drug-resistant epilepsy that was not limited to focal epilepsy but included idiopathic generalized epilepsies (IGE) and developmental and epileptic encephalopathies (DEEs).

## Materials and methods

### Study design

We performed a retrospective real-world study to investigate CNB usage in two German tertiary referral centers for epilepsy: Aachen University Hospital and Tübingen University Hospital. All patients who entered treatment with CNB for at least 2 weeks between October 2020 and June 2023 at either one of the study sites were analyzed. Patients were included regardless of their epilepsy diagnosis, i.e., also including patients with generalized epilepsies. We did not define a minimal dose requirement to capture early CNB discontinuations.

The primary outcome was early treatment response between 2 and 6 months of treatment. Secondary outcomes included response rates between months 6 and 11, and months 12 to 18 of treatment, response rates for generalized/focal to bilateral tonic–clonic seizures (GTCS/FBTCS) for all three time periods, adherence to CNB throughout the study, as well as the occurrence and nature of adverse drug reactions (ADRs) at 2, 6, 12, and 18 months. GTCS and FBTCS were grouped together for analyses due to low numbers of the GTCS group alone and due to shared features of both entities, e.g. an increased risk for sudden unexpected death in epilepsy and trauma, independently of their different etiology.

Baseline seizure frequency was extrapolated based on the reported seizure frequency by patients or caretakers during the previous 3 months. We defined seizure freedom as the total absence of seizures between two analysis time points. Treatment response was defined as a ≥ 50% seizure frequency reduction compared with baseline. We defined seizure worsening as an increase of > 100%. We defined four time points to consistently assess outcome parameters across the two study sites at 2, 6 12, and 18 months. We selected the 2-month time point because we expected the usual dosing schedule of up to 200 mg to be completed at that point and to assess early ADRs. We chose the remaining time points to align with the outpatient appointment policies of both sites.

A fixed dosing schedule was envisaged in all patients following official recommendations: 12.5 mg daily initial dose and increments at 2-week intervals to 25, 50, 100, 150, and 200 mg daily. However, dosing schemes could be adapted by the treating physicians if deemed necessary for tolerability reasons or if a meaningful response was reached earlier. Dose adjustments after attaining 200 mg per day were based on efficacy and tolerability considerations.

### Data collection and statistical analysis

We extracted clinical data from electronic medical records for baseline and each follow-up. Follow-ups could be more frequent than the analysis time points (e.g., 3 or 9 months after CNB initiation). If more than one follow-up occurred within one of the defined study intervals, we averaged the seizure frequency. We classified seizure and epilepsy types according to the current International League Against Epilepsy (ILAE) guidelines [16]. We also collected seizure etiology, previous and concomitant ASMs, and history of epilepsy surgery or neuromodulation. At each follow-up, patients were interviewed and examined for the occurrence of potential ADRs, changes of CNB dose, concomitant ASMs doses, and CNB discontinuation. We performed Chi-square tests to test the statistical significance of the association of treatment response with the number of previous ASMs. The significance level was set at *p* < 0.05.

### Ethics

This study was a retrospective analysis of existing clinical data and was approved by local ethic boards at both sites (Aachen: CTC-A 24-075; Tübingen: 167/2023BO2). Patient consent was waived due to the retrospective nature of the study.

## Results

### Cohort demographics

Between October 1, 2020, and June 30, 2023, 116 patients received treatment with CNB for at least two weeks and were included in the analysis. Baseline demographics are summarized in Table 1. While most patients had a diagnosis of focal epilepsy (85.3%), we also included patients with other diagnoses, such as IGE (3.4%), non-lesional Lennox-Gastaut-syndrome (LGS, 2.6%), and other types of DEE (8.6%). The cohort displayed a high prevalence of GTCS/FBTCS (85.3%). At baseline, the mean monthly seizure frequency was 29.7 (± 82.7, range 0–600); the frequency for GTCS/FBTCS was 1.6 (± 3.8; range 0–27.5).

**Table 1:** Demographics and baseline characteristics

Number of patients	116	
Age at CNB initiation, mean (range)	38.5	18–79
Female, *n* (%)	51	44.0%
Age of onset, mean (range)	13.1	0–59
Epilepsy syndrome, *n* (%)		
Focal epilepsy	99	85.3%
Idiopathic generalized epilepsy	4	3.4%
Lennox-Gastaut-syndrome	3	2.6%
Other developmental and epileptic encephalopathy*	10	8.6%
Seizure types, *n* (%)		
Focal aware	69	59.5%
Focal impaired awareness	93	80.2%
Generalized/bilateral tonic–clonic	99	85.3%
Other**	9	7.8%
*N* of ASM trials prior to CNB, median (range)	10.5	2–24
*N* of concomitant ASMs, median (range)	3	1–6
1 ASM, *n* (%)	12	10.3%
2 ASM, *n* (%)	39	33.6%
3 ASM, *n* (%)	42	36.2%
4 ASM, *n* (%)	18	15.5%
5 ASM, *n* (%)	4	3.4%
6 ASM, *n* (%)	1	0.9%
Type of concomitant ASMs at baseline, *n* (%)		
Brivaracetam	45	38.8%
Lamotrigine	40	34.5%
Valproic acid	37	31.9%
Perampanel	28	24.1%
Lacosamide	27	23.3%
Clobazam	23	19.8%
Cannabidiol	19	16.4%
Phenytoin	17	14.7%
Levetiracetam	14	12.1%
Topiramate	11	9.5%
Zonisamide	9	7.8%
Eslicarbazepine	9	7.8%
Other***	25	21.6%
Resective epilepsy surgery, *n* (%)	14	12.1%
VNS, *n* (%)	31	26.7%
DBS, *n* (%)	1	0.9%
Ketogenic diet, *n* (%)	4	3.4%

*ASM* Antiseizure medication; *IQR* interquartile range; *VNS* vagus nerve stimulation

*Dravet-Syndrome (2), tuberous sclerosis (2), GRIN2B-DEE (1), Rett-Syndrome (1), GABRB3-DEE (1), CHD2-DEE(1), BATF2-DEE (1), genetically unsolved DEE

**Myoclonic, absence, atypical absence, generalized tonic, generalized atonic

***Oxcarbazepine (5), phenobarbitone (4), pregabalin (3), gabapentin (3), carbamazepine (3), clonazepam (2), stiripentole (1), rufinamide (1), bromide (1), everolimus (1), medicinal cannabis (1)

At the time of CNB initiation, patients received a median of three concomitant ASMs (range 1–6). The top three concomitant ASMs were brivaracetam (38.8%), lamotrigine (34.5%), and valproic acid (31.9%). Including concomitant ASMs, patients had received a median of 10.5 ASMs prior to CNB initiation (range: 2–24). The cohort featured a high rate of failed epilepsy surgery (12.1%) and neuromodulation devices (27.6%).

### Patient retention

**Fig. 1:**
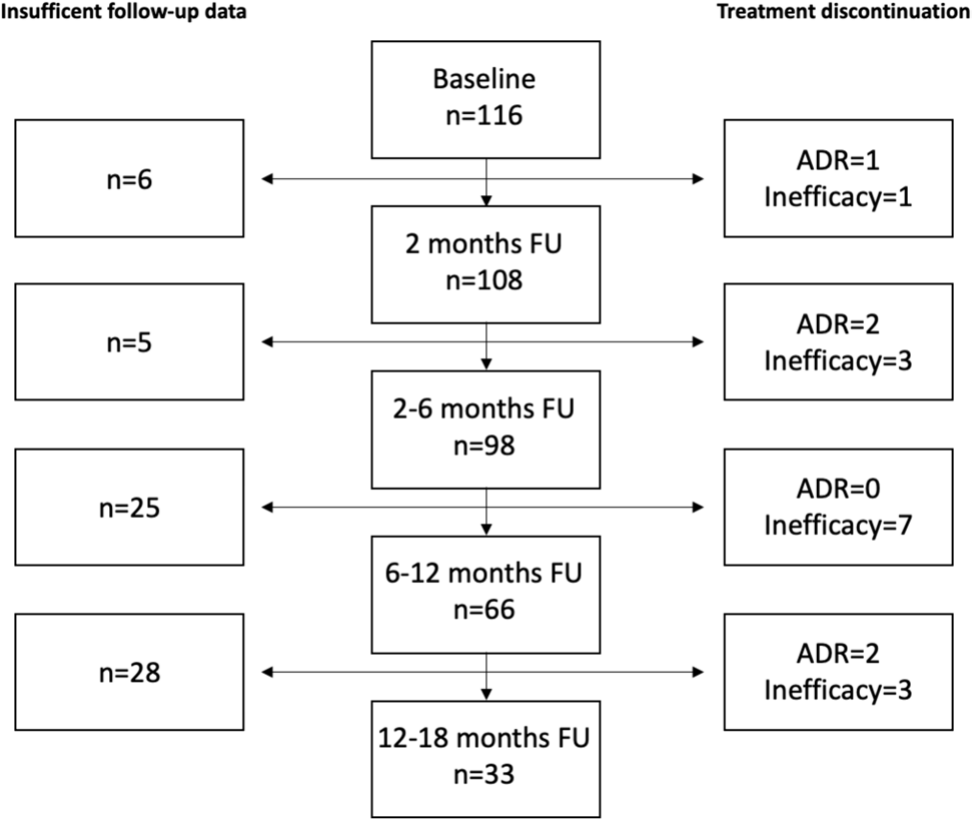
Study flow diagram. A total of 116 patients were included in the study being treated with cenobamate. Due to insufficient follow-up data, adverse drug reactions (ADRs) or inefficacy treatment was discontinued at different time points and patients could not be considered for all analyses. *FU* follow-up

Of 116 patients included in the analysis, 108 had sufficient follow-up data at 2 months, 98 for the period of 2 to 6 months, and 66 and 33 for the following two observation periods (Fig. 1). Overall, 14 patients stopped CNB because of insufficient treatment response and 5 patients due to ADRs. The median dose of CNB at the last follow-up was 200 mg (interquartile range, IQR 100 mg; range 25–400 mg) with a median treatment duration of 12 months (IQR 7–18 months, range 1–27 months).

### Treatment response

**Fig. 2:**
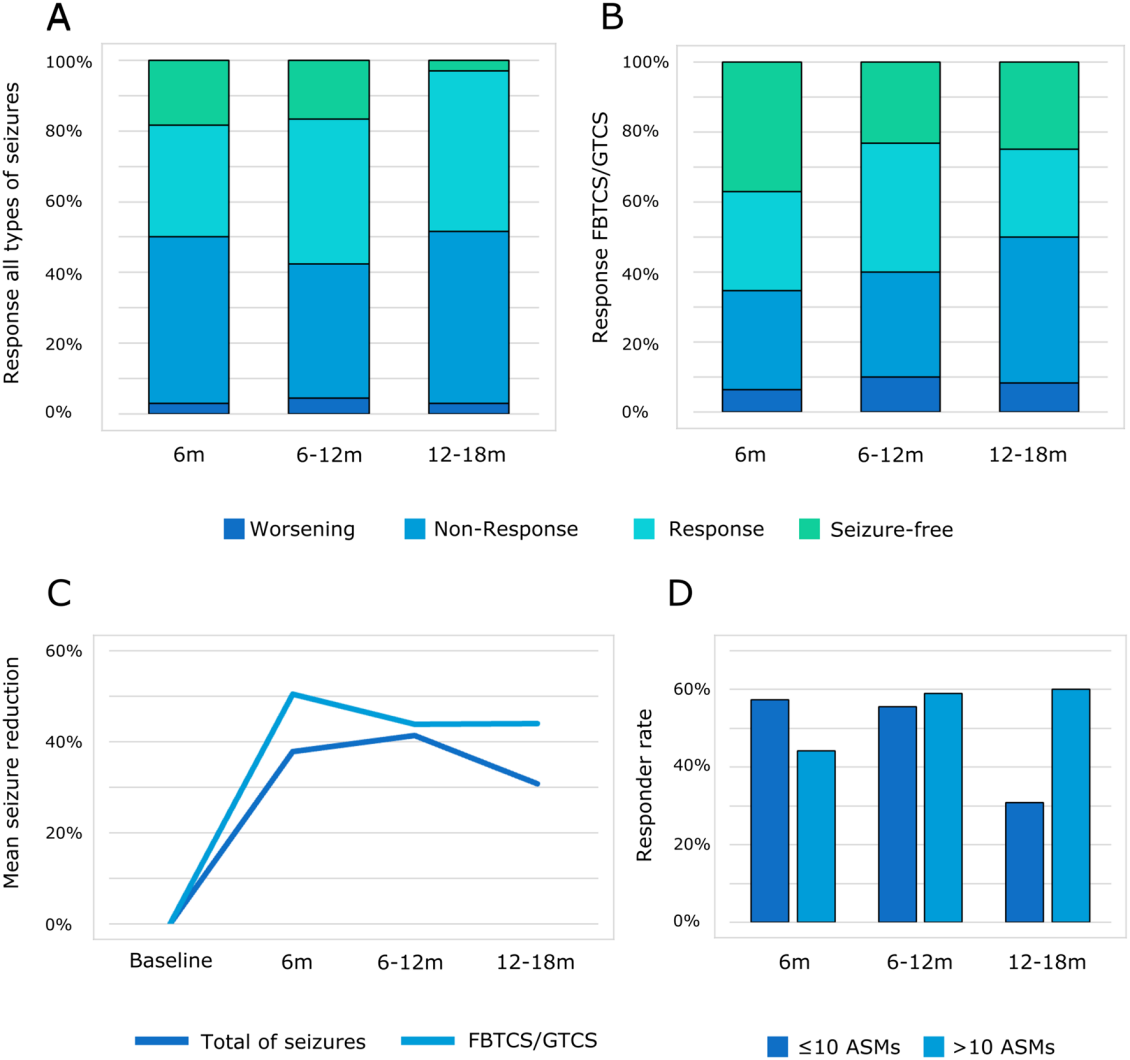
Cenobamate treatment response in % seizure reduction to baseline **A** including all types of seizures **B** including focal to bilateral and generalized tonic–clonic seizures (FBTCS/GTCS). Showing on the left treatment response after 2–6 months, in the middle after 12 and more months and on the right side after 18 and more months. Colors: green for seizure-freedom since treatment with CNB, turquoise for response defined as > 50% seizure reduction, blue for non-response and dark blue for seizure worsening. **C** Mean seizure reduction over time in % (at baseline, six months, six to 12 months, and 12 to 18 months). Colors: dark blue for total of seizures, blue for FBTCS and GTCS. **D** Responder rates (in %) did not differ significantly in the two groups with ten or less (dark blue) and more than ten (blue) antiseizure medications (ASMs) over time (six months, six to 12 and 12 to 18 months)

At 6 months, 50% (49/98) of patients were responders to CNB, i.e., they displayed a seizure reduction of all seizures ≥ 50% compared to baseline. For the intervals up to 12 and 18 months, we found no relevant change in response rates: 57.5% (38/66) and 48.5 (16/33), respectively (Fig. 2A). Seizure frequency compared with baseline declined by 37.9%, 41.3%, and 30.8% for the 6-, 12-, and 18-month period, respectively (Fig. 2C). Seizure freedom was achieved in 18.4% (18/98) patients at 6 months, in 16.7% (11/66) up to 12 months, and in 3.0% (1/33) up to 18 months (Fig. 2A).

46 patients experienced GTCS/FBTCS at baseline and were eligible for the 6-month follow-up. Response rates for generalized/bilateral tonic–clonic seizures were more elevated, with 65.2% (30/46) at 6 months, 60.0% (18/30) for up to 12 months, and 50.0% (6/12) for up to 18 months (Fig. 2B). Seizure frequency of GTCS/FBTCS compared with baseline declined by 50.5%, 43.8%, and 44.0% for the 6-, 12-, and 18-month periods, respectively (Fig. 2C). Seizure freedom for GTCS/FBTCS was achieved in 37.0% (17/46) at 6 months, in 23.3% (7/33) up to 12 months, and in 25.0% (3/12) up to 18 months (Fig. 2B).

We then tested whether treatment response was associated with the number of failed ASMs and selected > 10 ASMs as cut-off based on the median number of previous ASMs. Response rates were 57.4 vs. 44.2% for individuals with ≤ 10 vs. > 10 previous ASMs at 6 months and not significantly different; *χ*^2^(1, *N* = 99) = 1.72, *p* = 0.19. Likewise, response rates at 12 months; 55.6 vs. 59.0%, *χ*^2^(1, *N* = 66) = 0.07, *p* = 0.778, and at 18 months; 30.8 vs. 60.0%, *χ*^2^(1, *N* = 33) = 2.70, *p* = 0.10, did not differ significantly. (Fig. 2D). The rate of seizure freedom appeared more elevated in individuals with ≤ 10 ASMs at 6 months (25.5 vs. 13.6%) but did not differ at 12 months (14.8 vs. 11.9%), and 18 months (0 vs. 1.7%).

### Adverse drug reactions

The most common ADR was fatigue, reported by 30.1% of individuals (Table 2). ADRs often observed in conjunction with sodium channel inhibitors, such as dizziness, gait disturbance, tremor, and double vision, were also reported in our cohort but were usually not a reason to discontinue CNB. Only 9 individuals (7.7%) stopped CNB due to ADRs, thereof 4 later than 18 months after CNB initiation. Extreme fatigue and weight loss were most common. One patient in their 20 s stopped CNB because of the occurrence of suicidal ideations 2 months after starting CNB. This person had a history of depression and anxiety disorder. After discontinuing CNB and regular psychological follow-ups, suicidal ideations resolved. There was no case of drug rash with eosinophilia and systemic symptoms (DRESS) or other notable skin reactions.

**Table 2:** Adverse effects listed according to their frequency in *n* = absolute patient numbers (% of all patients)

Adverse drug reaction	Total number reported	Main reason for discontinuation
Fatigue	35 (30.1%)	3
Dizziness	16 (13.8%)	
Gait disturbance	6 (5.2%)	
Mood changes	5 (4.3%)	1
Nausea	4 (3.4%)	
Slurred speech	4 (3.4%)	
Decreased appetite, weight loss	3 (2.6%)	2
Tremor	2 (1.7%)	
Double vision	2 (1.7%)	
Memory difficulties	2 (1.7%)	1
Suicidal ideation	1 (0.9%)	1

### Cenobamate concordance after 18 months

**Fig. 3:**
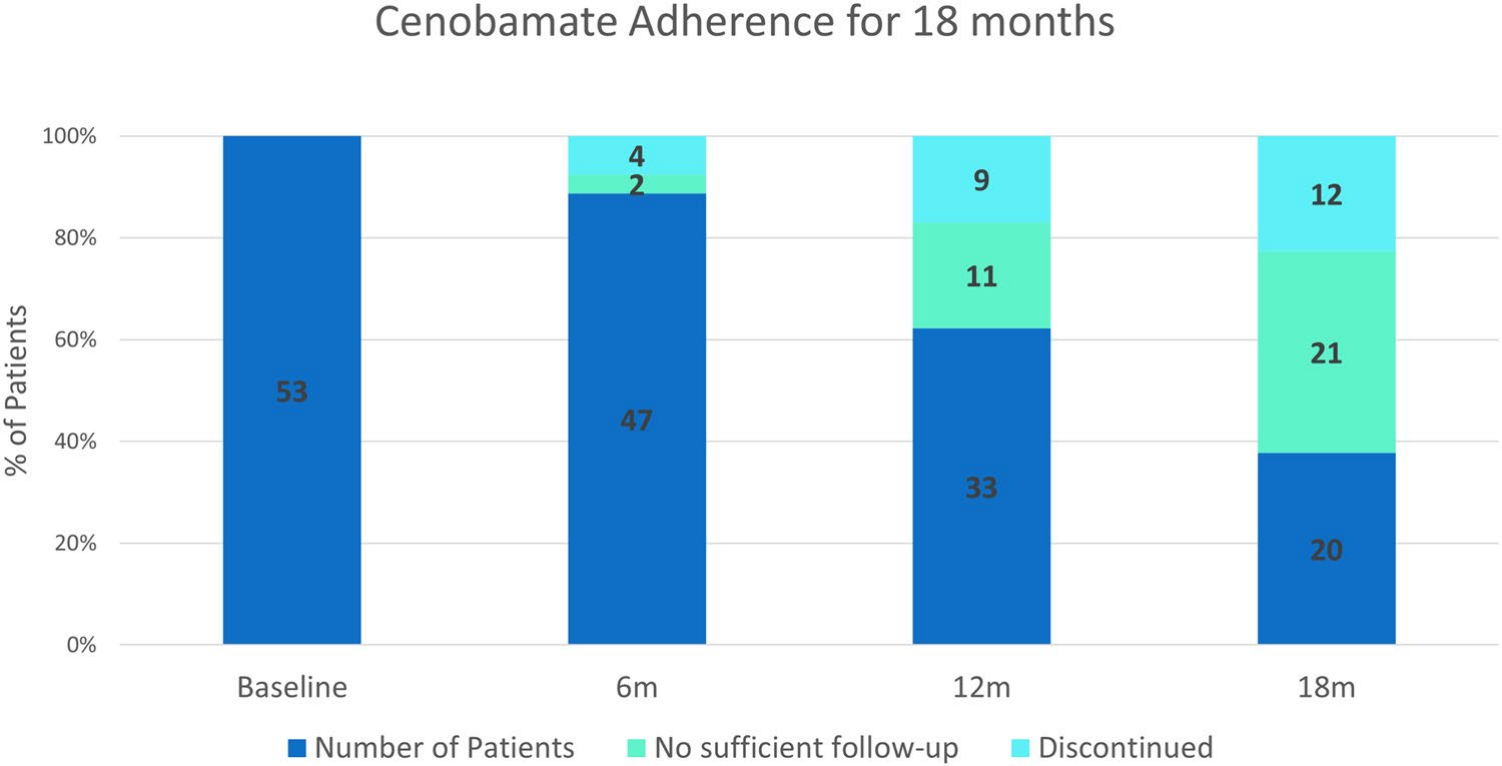
Cenobamate (CNB) concordance for all patients initiated treatment before January 1, 2022, who could have been treated for 18 months. Dark blue indicates: number of patients still on CNB treatment; Turquoise indicates: patients who discontinued CNB treatment due to side effects or ineffectiveness. Green indicates: patients for whom there were no further follow-ups

In order to have an overall assessment of tolerability and efficacy, we were interested in the number of patients who could have been treated with cenobamate for 18 months. Therefore, we evaluated all patients who started treatment with CNB before January 1, 2022 (53 patients). Follow-up data were available for 32 of 53 patients (more than 60%), but no reliable statement can be made for 21 patients because no specific contact was made with patients in our retrospective real-world setting. However, at least 20 patients (37.7%) were still being treated with CNB after 18 months and only 12 patients (22.6%) discontinued it (Fig. 3).

#### Subsequent changes of concomitant anti-seizure medications

After initiating CNB, we found that at least one concomitant ASM was stopped in 58.6% (68/116) of cases during the observation period. A dose reduction of at least one ASM was performed in 44.0% (51/116). The initiation of additional ASMs during the observation period occurred in 20.7% (24/116), a dose escalation of at least one concomitant ASM in 11.2% (13/116) of cases.

**Fig. 4:**
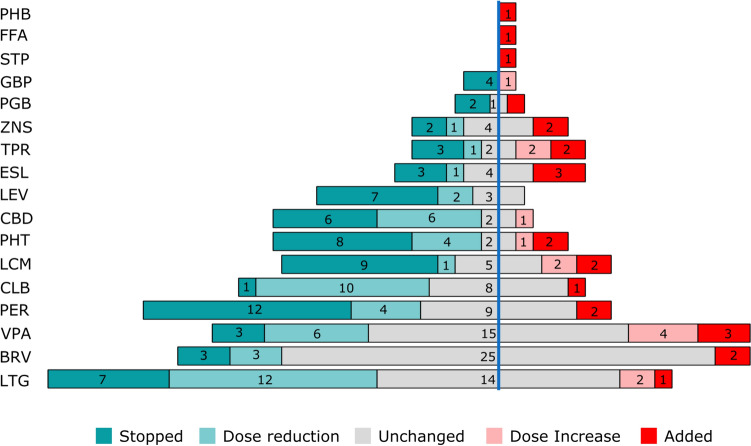
Change of concomitant medication. Patients had 17 different concomitant antiseizure medications (ASMs), which were in a great majority of patients (213/250) unchanged (grey, *n* = 94) or even reduced (green, *n* = 51) or stopped (dark green, *n* = 68). Increases of doses (light red, *n* = 13) and an addition of medication were rarer (*n* = 26). Lamotrigine (LTG, *n* = 36), brivaracetam (BRV, *n* = 33) and valproic acid (VPA, *n* = 31) were the most common drugs in combination in our cohort. Other drugs were lacosamide (LCM), cannabidiol (CBD), perampanel (PER), phenytoin (PHT), topiramate (TPR), levetiracetam (LEV), clobazam (CLB), eslicarbazepine (ESL), pregabalin (PGB), gabapentin (GBP), zonisamide (ZNS), stiripentol (STP), fenfluramine (FFA), phenobarbital (PHB)

The list of ASMs that were most often discontinued was headed by perampanel (*n* = 12), lacosamide [n=9], and phenytoin [n=8]. Dose reduction was most frequently performed for lamotrigine [n=12], clobazam [n=10], and cannabidiol and valproic acid (both n=6). The most frequently added new concomitant ASMs were eslicarbazepine [n=3] and valproic acid [n=3] (Fig. 4).

Of the five most common concomitant ASMs, brivaracetam was the drug that remained at a stable dose most frequently (75.8% of all subjects receiving brivaracetam) compared with valproic acid (48.4%), lamotrigine (38.9%), perampanel (33.3%), and lacosamide (26.3%) (Fig. 4).

## Discussion

Our study is, to our knowledge, one of the largest CNB real-world analyses so far [13, 17]. In analogy to smaller real-world studies, we found CNB to be an efficient ASM with a good treatment response rate, an unusual seizure freedom rate in drug-resistant patients, and a favorable tolerability profile [12–15, 17]. Unlike most previous studies, about a third of patients had an observation of 18 months or longer [12, 13].

A long disease history characterized our patient cohort. With a median of 10.5 precedent ASM trials and 3 concomitant ASMs at the time of CNB initiation, ASM profiles were comparable to previous real-world studies [12–15], mirrored by a high prevalence of epilepsy surgery and neuromodulation. The composition of concomitant ASMs depicted a high usage of relatively novel ASMs, such as brivaracetam, perampanel, or off-label use of cannabidiol, as well as frequent use of third-line ASMs, such as phenytoin, clobazam, and valproic acid, underlining that patients were at the end of a long treatment journey.

Given the composition of our patient cohort, we observed a high responder rate of about 50% and a high seizure freedom rate of 18%. Interestingly, the number of previous ASM trials did not influence the outcome, emphasizing that CNB is a compelling choice in ultra-resistant epilepsy patients. However, our responder rate appears somewhat lower than in a previous meta-analysis of real-world studies, including adult and pediatric patients [17] and other more recent studies [11–13], reporting responder rates between 55 and 68%. This observation might be related to a larger share of patients with DEE in our cohort, who are more prone to severe drug resistance. Nonetheless, seizure freedom rates in our cohort and response rates for GTCS/FBTCS aligned with those reported previously [11–13, 17]. Interestingly, we noticed a tendency for the seizure freedom rate and the percentual seizure reduction to decline in our latest observation period, whereas the responder rate remained stable. Whether this observation hints at a long-term decrease in CNB efficiency, an effect that has been described for other ASMs [18], is limited by our reduced sample size for this period and remains to be elucidated in long-term observational studies. Even though the comparison with real-world data for other of the more recent ASMs, such as perampanel and brivaracetam, is flawed due to differently composed patient cohorts, ours and previous efficacy results appear somewhat more favorable for CNB: e.g., for perampanel, in a large observational cohort, the 6-months > 50% responder rate was reported at 42%, the 1-year retention rate at 48% [19]; for brivaracetam, an extensive pooled analysis > 50% response at 6 months was achieved in 32% of patient with one-year retention of 71% [20].

The quality and quantity of ADRs in our cohort were comparable with previous real-world studies [11–13] and aligned with the initial phase III randomized controlled trials (RCTs) [8, 10]. Overall, the degree of ADRs was mild to moderate in most cases. We observed weight loss in three patients, a potential ADR that has not been the focus of previous reports. However, weight loss or decreased appetite was reported in a few cases in a phase III RCT [8] and several real-world studies [11, 13, 14]. In two of our patients, it led to treatment discontinuation with subsequent normalization of body weight. A more stringent appreciation of changes in body weight of patients treated with CNB could help better evaluate this potential ADR in the future. In another patient, CNB was stopped due to the occurrence of suicidal ideations in a patient in their 20 s with focal epilepsy and a history of anxiety disorder and depression. Whether the occurrence of the suicidal ideations was related to CNB is questionable and could also be associated with the patient’s history of psychiatric disorders. Overall, the frequency of psychiatric ADRs in our cohort was low and reflects previous studies that report low rates of psychiatric ADRs [13, 21]. Other severe ADRs were not observed, and treatment discontinuation due to ADRs was rare, supporting an overall favorable tolerability profile of CNB. This is further supported by the fact that a higher number of patients remained on CNB after 18 months compared to those who had discontinued. The good tolerability data could also be partly due to the limited use of sodium channel blockers, in particular the low proportion of patients with carbamazepine in the concomitant medication.

The addition of CNB entailed further changes in concomitant ASMs in most patients. One obvious adaptation was reducing or discontinuing sodium channel inhibitors such as phenytoin, lamotrigine, lacosamide, and eslicarbazepine. Whereas the pharmacokinetic interaction between CNB and phenytoin is well-known, frequently resulting in a relevant and actionable augmentation of phenytoin plasma levels [10, 22, 23], it is unclear whether the reduction of other sodium channel inhibitors was warranted. A post hoc analysis of the RCT C017 showed no significant differences in the rate of ADRs between combinations of CNB with or without sodium channel blockers [24]. Another frequently encountered change in our cohort was the dose reduction of clobazam. In analogy to phenytoin, CNB inhibition of CYP2C19 leads to an increase of the active metabolite of clobazam: *N*-desmethyl clobazam and reduction of clobazam is generally advised [23, 25]. We also found that perampanel was discontinued in many patients. We are unaware of any direct interactions but postulate that prescribers anticipated an increased risk of dizziness and balance problems that are common ADRs for both ASMs. We also assume that perampanel was the last drug initiated prior to CNB and was then exchanged due to insufficient efficacy. Brivaracetam, the most common concomitant ASM in our cohort, however, was kept at a stable level, suggesting that tolerability issues between the two drugs were rare and that prescribers anticipated no relevant interactions.

Our retrospective, observational study design implies several limitations, such as a heterogeneous cohort, potential deviations from the usual CNB dosing recommendations, off-label use in patients with generalized seizures, and the lack of a control study arm to assess the impact of placebo effects that could have been nurtured by positive expectations by prescribers and patients. Moreover, no data were available for the later follow-up periods in many cases, limiting conclusions about long-term effects. Additionally, all data sets were exclusively collected from German centers, which could possibly lead to a different country-specific use of ASMs. However, our results align with previous studies and corroborate their findings.

In conclusion, CNB proves to be an efficient and, in general, well-tolerated ASM. Especially in ultra-drug-resistant epilepsy patients, CNB appears to provide response and even seizure freedom rates that seem unmet by other novel ASMs. Considering dose adaptation recommendations for certain ASMs, e.g., phenytoin and clobazam, CNB also appears to be a pertinent and well-tolerated ASM in multi-drug combination schemes.

## Data Availability

Data is available upon request.
