# Correction to: Cenobamate: real-world data from a retrospective multicenter study

**DOI:** 10.1007/s00415-024-12606-8

**Published:** 2024-08-21

**Authors:** Stephan Lauxmann, David Heuer, Jan Heckelmann, Florian P. Fischer, Melanie Schreiber, Elisabeth Schriewer, Guido Widman, Yvonne Weber, Holger Lerche, Michael Alber, Sigrid Schuh-Hofer, Stefan Wolking

**Affiliations:** 1 Department of Neurology and Epileptology, Hertie Institute for Clinical Brain Research, University of Tuebingen, Hoppe-Seyler-Str. 3, 72076 Tuebingen, Germany; 2 Department of Epileptology and Neurology, RWTH Aachen University Hospital, Aachen, Germany; 3 Department of Pediatric Neurology and Developmental Medicine, University Children’s Hospital, Tuebingen, Germany

**Correction to: Journal of Neurology** 10.1007/s00415-024-12510-1

In the original version of this article, abstract was incorrect and should have been

**Abstract**

**Background** Clinical trials have shown that cenobamate (CNB) is an efficacious and safe anti-seizure medication (ASM) for drug-resistant focal epilepsy. Here, we analyzed one of the largest real-world cohorts, covering the entire spectrum of epilepsy syndromes, the efficacy and safety of CNB, and resulting changes in concomitant ASMs.

**Methods** We conducted a retrospective observational study investigating CNB usage in two German tertiary referral centers between October 2020 and June 2023 with follow-up data up to 27 months of treatment. Our primary outcome was treatment response. Secondary outcomes comprised drug response after 12 and 18 months, seizure freedom rates, CNB dosage and retention, adverse drug reactions (ADRs), and changes in concomitant ASMs.

**Results** 116 patients received CNB for at least two weeks. At 6 months, 98 patients were eligible for evaluation. Thereof 50% (49/98) were responders with no relevant change at 12 and 18 months. Seizure freedom was achieved in 18.4% (18/98) at 6 months, 16.7% (11/66), and 3.0% (1/33) at 12 and 18 months. The number of previous ASMs did not affect the seizure response rate. Overall, CNB was well-tolerated, however, in 7.7% (9/116), ADRs led to treatment discontinuation. The most frequent changes of concomitant ASMs included the discontinuation or reduction of sodium channel inhibitors, clobazam reduction, and perampanel discontinuation, while brivaracetam doses were usually left unchanged.

**Conclusions** CNB proved to be a highly effective and generally well-tolerated ASM in patients with severe drug-resistant epilepsy, comprising a broad array of epilepsy syndromes beyond focal epilepsy.

The original article has been corrected.

